# Primary squamous cell carcinoma of the endometrium: A case report

**DOI:** 10.1097/MD.0000000000045920

**Published:** 2025-11-14

**Authors:** Shuhui Cui, Zhe Dong

**Affiliations:** a Obstetrics and Gynecology Department, Peking University International Hospital, Beijing, China.

**Keywords:** endometrium carcinoma, prognosis, squamous carcinoma, treatment

## Abstract

**Rationale:**

Primary squamous cell carcinoma of the endometrium (PSCCE) has a low incidence and is relatively rare. The treatment for this disease is currently not well-defined. Here, we report a case of a 56-year-old female patient. Based on a review of previous literature, we summarized the treatment options for this rare disease and presented our insights.

**Patient concerns:**

A 55-year-old patient presented with postmenopausal vaginal bleeding. Transvaginal ultrasonography demonstrated uterine cavity hydrocele and abnormal echo. Diagnostic hysteroscopy was performed, and pathological results revealed PSCCE.

**Diagnosis:**

The patient was diagnosed with primary squamous cell carcinoma of the endometrium (stage IA).

**Interventions:**

The patient underwent laparoscopic total hysterectomy and bilateral adnexectomy, combined with pelvic and para-aortic lymph node dissection. Then she received external beam radiotherapy and brachytherapy.

**Outcomes:**

The patient has been undergoing regular follow-up and so far, no recurrence of the tumor has been detected.

**Lessons:**

Primary squamous cell carcinoma of the endometrium is rare, no definitive treatment protocol has been established. Early diagnosis is important for prognosis.

## 1. Introduction

Primary squamous cell carcinoma of the endometrium (PSCCE) is a rare subtype of endometrial malignancy first documented in 1892. The literature searched up to March 2025 were all case reports, and there were only over 100 cases at present. The largest series includes 8 cases reported by Goodman et al^[1]^ in 1996 and Horn et al^[2]^ in 2006. According to the World Health Organization Classification of Tumors of the Female Reproductive Organs (5th Edition),^[3]^ PSCCE accounts for <5% of all endometrial cancers. Here, we present a case of PSCCE.

## 2. Case report

**Figure 1.**
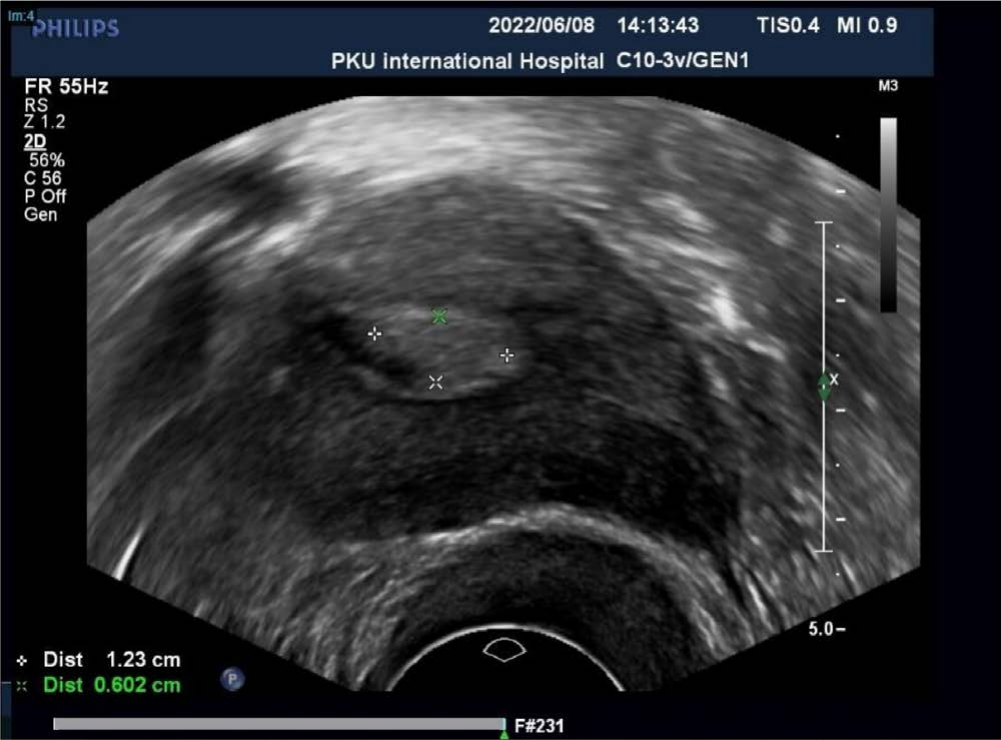
Transvaginal ultrasonography revealed a space-occupying lesion measuring 12.3 × 6 mm in the uterine cavity.

**Figure 2.**
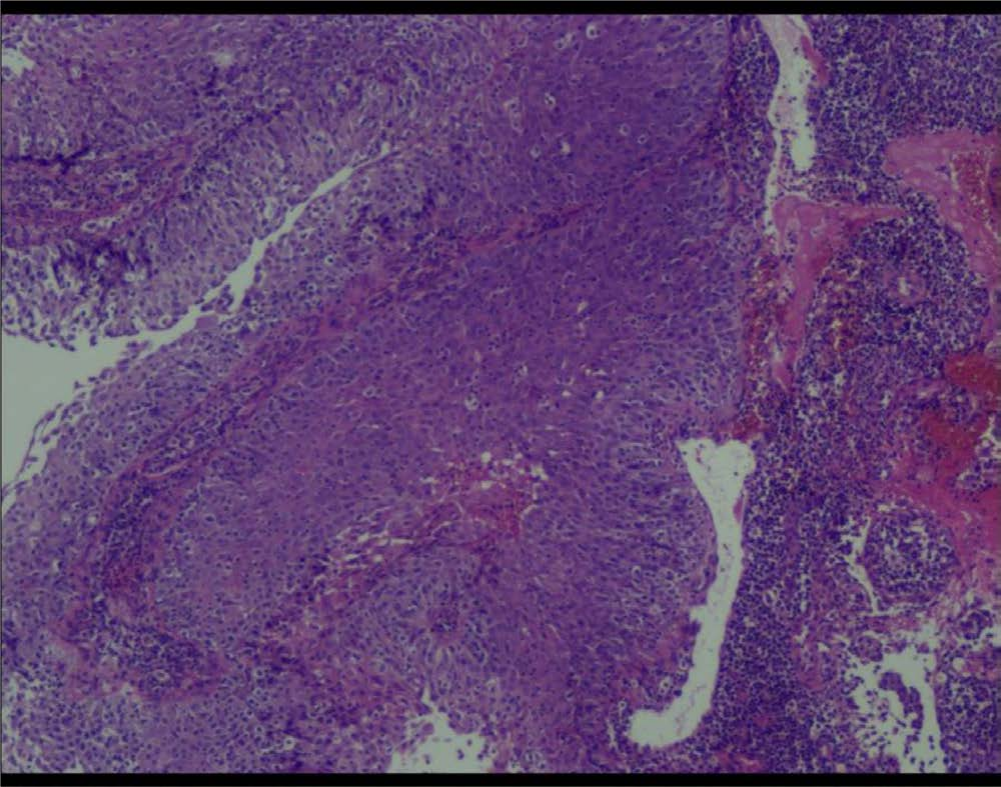
Histopathology of the endometrium (Hematoxylin–eosin staining).

A 55-year-old postmenopausal woman, gravidity 3 and parity 1, was presented to gynecological clinic with the history of a small amount of vaginal bleeding. She had been postmenopausal for 7 years and had no vaginal bleeding prior to her recent symptoms. She denied any past medical history and a family history of malignant tumors. No visible signs of abnormality during the bimanual examination. The human papillomavirus (HPV) and Thin Prep cytologic test of the cervix were negative. Transvaginal ultrasonography demonstrated thin endometrium, uterine cavity hydrocele and abnormal echo (Fig. 1). Hysteroscopy was performed simultaneously with diagnostic curettage and focal excision. Pathological results revealed poorly differentiated squamous cell carcinoma of the endometrium, accompanied by lamellar necrosis (Fig. 2). Immunohistochemical showed positive for p40, p63, MSH6, MSH2, MLH1, PMS2, P53 (mostly +), Ki-67 (about 60%+), Vimentin (partial +), PAX-8 (partial +), WT1 (partial +), CK5/6 (partial +), ER (locally weak +), PR (focal +), and negative for p16, CK7. Some of the immunohistochemical indicators are used to confirm the diagnosis of endometrial squamous cell carcinoma. High expression of p40 and p63 is closely associated with the occurrence of squamous cell carcinoma. CK5/6 is positive in squamous epithelial cells and negative in glandular cells. The CK7 marker is associated with glandular epithelium and is typically expressed in adenocarcinomas. Whether P16 is positive indicates whether the tumor is related to HPV infection. Vimentin is expressed in most soft tissue tumors and also shows positive reactions in some epithelial tumors. WT1 helps us in the process of differential diagnosis. The PAX-8 marker is applicable to all uterine, cervical and ovarian tumors of Mullerian origin, and it has been indicated as positive in previous case reports. Therefore, we conducted this test. Some of the immunohistochemical indicators are used to indicate the prognosis of the disease. A positive P53 result usually indicates a more aggressive nature and a poorer prognosis. Ki-67 is mainly used to assess the proliferative activity of tumor cells. A positivity rate of ≥ 30% is often associated with active tumor cell proliferation and strong invasiveness, and the prognosis may be poorer. Some of the immunohistochemical indicators are used to guide the treatment. Positive ER/PR indicates that the tumor is responsive to endocrine therapy. By detecting the 4 known MMR proteins, MSH6, MSH2, MLH1, and PMS2, we can determine whether there is a deficient mismatch repair. If there is a deficient mismatch repair, PD-1 antibody therapy can be adopted.

Due to this pathological finding, the patient was admitted to our hospital again 2 weeks later. Contrast-enhanced computed tomography of the whole chest, abdomen and pelvic showed no positive signs. No enlarged lymph nodes were found retroperitoneally. Pelvic enhanced magnetic resonance imaging also found no abnormal findings. Considering that the lesion is confined to the uterus, with no involvement of the cervix. The uterus is of normal size and the vagina shows no significant narrowing. So we evaluated that the uterus could be completely removed through the vagina. Therefore, the patient underwent laparoscopic total hysterectomy and bilateral salpingo-oophorectomy, combined with pelvic and para-aortic lymph node dissection. On gross examination, the uterus and bilateral adnexa appeared normal. The pathological results showed that no carcinoma remained in the endometrium. No cancer was found in the cervix or parauterine tissue. 29 pelvic lymph nodes and 7 para-aortic lymph nodes were removed, and no tumor metastasis was found. No tumor cells were found in the peritoneal washings.

According to the Figo 2009 staging system, the patient is in stage IA. On account of the special pathological type and the poor differentiation of the tumor, the patient was then transferred to department of radiotherapy for external beam radiotherapy and brachytherapy. The patient was followed up regularly. During the 36-month period after the completion of the treatment, the patient regularly underwent tumor marker tests and imaging examinations. No recurrence or metastasis has been reported to date.

## 3. Discussion

Primary squamous cell carcinoma of the endometrium (PSCCE) is a rare subtype of endometrial malignancy. Currently, the etiology, clinicopathological features, treatment strategies, and prognosis of PSCCE remain poorly understood.

Due to the low incidence rate of PSCCE, only 1 case was reported in our article. Therefore, we do not have a large amount of case data for analysis, which limits the scope of our study. However, we still summarized some of the cases that have been reported up to now, in order to explore the causes, clinical manifestations, treatment methods and prognosis of PSCCE.

Potential origins of PSCCE cancer cells include differentiation of endometrial precursor cells, squamous metaplasia of endometrial cells, and ectopic cervical tissue.^[4]^ Various factors, such as pyometra, uterine prolapse, low estrogen levels, intrauterine device use and chronic inflammation, may induce the differentiation of endometrial precursor cells into squamous epithelial cells, thereby contributing to the development of PSCCE.^[5]^ In addition, case reports^[6,7]^ have suggested an association between PSCCE and uterine ichthyosis. At present, there is no consensus regarding the role of human papillomavirus (HPV) infection in the pathogenesis of PSCCE. In our case, the HPV test was also negative. While some studies have reported an association between HPV infection and PSCCE,^[8,9]^ the majority of cases are not related to HPV infection.^[10]^

From a molecular pathology perspective, several reports^[4]^ have indicated a possible link between PSCCE and p53 gene mutations. However, Horn et al^[2]^ conducted an immunohistochemical analysis on 8 cases of PSCCE, finding only one case positive for p53, while 4 cases were positive for p16. Fanni et al^[11]^ first reported a case of PSCCE that tested positive for PAX-8 via immunohistochemical analysis, indicating the potential Mullerian origin of PSCCE. Due to the limited number of cases, the precise molecular pathological characteristics of PSCCE remain unclear. The immunohistochemical results of the case reported in our paper showed that P16 was negative and p53 and PAX-8 were positive. Furthermore, immunohistochemistry demonstrates that PSCCE expresses squamous differentiation markers such as P40, P63, and CK5/6.^[12]^ Our case also showed the same result.

Clinically, PSCCE predominantly occurs in perimenopausal and postmenopausal women. Yamashina and Kobara^[13]^ summarized 29 previously reported cases, with an average age of 61 years, which is 3–5 years older than patients with endometrial adenocarcinoma. The incidence in young women is extremely rare, with only 3 cases reported globally.^[8,10,13]^ The clinical manifestations of PSCCE are similar to those of endometrial adenocarcinoma and lack specificity. Postmenopausal vaginal bleeding is the most common symptom.

Fluhmann proposed diagnostic criteria for PSCCE^[5]^: no coexisting glandular carcinoma in the endometrium; no connection between the tumor in the endometrium and the squamous epithelium of the cervix; no primary squamous cell carcinoma of the uterus. Furthermore, in 1975, the World Health Organization added a supplementary condition^[5]^: tumor tissue must exhibit keratinized pearls or intercellular bridge structures. Preoperative diagnosis of PSCCE remains challenging, pathologic examination after hysterectomy is still required to confirm the diagnosis.^[14]^

Goodman et al^[1]^ reported that among 64 cases of PSCCE (including 56 cases from the literature), the median time from symptom onset to diagnosis was 11.5 months, underscoring the lack of effective screening and early diagnostic measures for this disease.

Regarding treatment, there is currently no unified consensus on the management of PSCCE. Treatment primarily involves surgery, followed by adjuvant radiotherapy and/or chemotherapy^[15]^

The basic surgical procedure for PSCCE involves total hysterectomy combined with bilateral salpingo-oophorectomy. Regarding the choice of surgical approach, open abdominal surgery was traditionally utilized, while laparoscopic techniques have been increasingly reported in recent years.^[7,11]^

Given the unclear lymph node metastasis rate of PSCCE, pelvic, para-aortic, and inguinal lymphadenectomy may be performed based on tumor invasion depth and metastatic status. Postoperative external pelvic radiation therapy can also be considered if necessary. Chemotherapy has been shown to reduce long-term recurrence and enhance tumor cell sensitivity to radiation, thereby improving the efficacy of radiotherapy. Kennedy et al^[15]^ first reported adjuvant cisplatin-based chemotherapy following PSCCE treatment in 1995, and most current literature supports platinum-based regimens. Aside from surgery combined with chemoradiotherapy, no alternative treatment options have been documented. Due to the rarity of PSCCE in women of childbearing age, fertility-sparing therapies have not been explored.

The prognosis of PSCCE is generally poorer than that of endometrial adenocarcinoma,^[16]^ and it is closely associated with surgical pathological staging. Patients at stages I and II tend to have better prognosis compared to those at stages III and IV. A few stage III patients who received postoperative radiotherapy combined with chemotherapy remained alive after a 10-year follow-up period. The primary causes of death were tumor recurrence and metastasis, with pelvic and abdominal metastases being the most prevalent, followed by lung, brain, and bone metastases.^[5]^ The patient reported in our case report was stage IA, and she received the operation and postoperative radiotherapy. The patient was still alive after 30-month follow-up.

## 4. Conclusion

In summary, PSCCE is a rare malignant endometrial tumor, and its etiology and pathogenesis remain unclear. Due to its low incidence and limited number of reported cases, no definitive treatment protocol has been established. Prognosis is closely associated with pathological staging, underscoring the importance of early diagnosis and timely intervention in extending survival.

## Author contributions

**Conceptualization:** Zhe Dong.

**Writing – original draft:** Shuhui Cui.

**Writing – review & editing:** Zhe Dong.
